# Squamous odontogenic tumor-like proliferation in a radicular cyst: A case report

**DOI:** 10.4317/jced.51056

**Published:** 2013-12-01

**Authors:** Sergi Sala-Pérez, Vicente Marco-Molina, Cosme Gay-Escoda

**Affiliations:** 1 DDS. Master of Oral Surgery and Implantology. Barcelona University Dental School. Spain; 2 MD, PhD. Head of the Service of Pathology. Quirón Hospital, Barcelona. Consultant pathologist of the Teknon Medical Center and Catalunya General Hospital. Barcelona. Spain; 3 MD, DDS, PhD. Chairman of Oral and Maxillofacial Surgery. Director of the Master of Oral Surgery and Implantology. University of Barcelona Dental School. Coordinating investigator of the IDIBELL Institute. Head of the Service of Maxillofacial Surgery, Teknon Medical Center. Barcelona, Spain

## Abstract

The squamous odontogenic tumour is a rare benign neoplasm whose aetiology remains unknown. It usually appears in the jaw and its origin could be related to the ephitelial remnants of Malassez. Histologically comprises numerous islets of squamous, non-keratinized, well-differentiated and rounded epithelial cells a fibrous stroma without signs of atypical cells. There is a non-neoplastic lesion with the same histological pattern than the squamous odontogenic tumour. This entity is characterized by squamous odontogenic tumour proliferations isolated into the cyst wall of an odontogenic cyst. It is rare and has a benign behavior. It has been suggested that these epithelial proliferations could be the former expression of the neoplastic form. It is very important to carry out clinical and radiological controls periodically. So far it has not been documented any change towards a squamous odontogenic tumour nor toward malignancy in a squamous odontogenic tumour like proliferation.

** Key words:**Radicular cyst, squamous odontogenic tumour.

## Introduction

The squamous odontogenic tumour (SOT) is a rare and benign neoplasm frequently located within the jaws. In 1975, Pullon et al. (1) identified this entity and reported it for the first time in a series of 6 cases. This benign tumour has a slow and gradual growth that might invade the trabecular bone, destroying the cortical bone and infiltrating adjacent structures (2). Histologically it is formed by numerous islands of squamous, non-keratinized, well-differenciated and rounded epithelial cells scattered in a fibrous tissue stroma with no signs of cellular atypia (3). Its aetiology remains unknown although it could be originated from the epithelial remnants of the Malassez. It usually appears over the lateral radicular surface of an erupted tooth and diminishes the height of alveolar bone causing tooth mobility (4). There is a similar entity that is characterized by squamous odontogenic tumour like proliferations (SOTLP) with a very similar histological pattern than the SOT. This lesion commonly is located in the wall of an odontogenic cyst and has a non-neoplastic character like in the SOT, representing probably, an hamartomatous lesion(5). Herein, we present a case of a SOTLP emerging in a radicular cyst of the maxilla.

## Case Report

**Figure 1:**
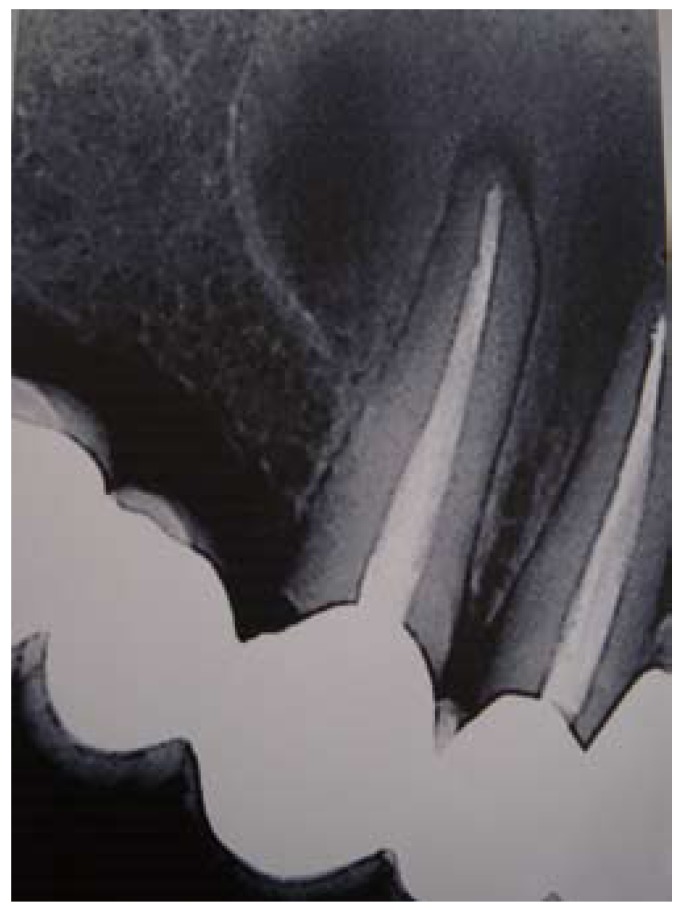
Periapical X-ray view. Radiotransparency in the anterior zone of the right upper maxilla, enveloping teeth #6, #7 and #8.

**Figure 2:**
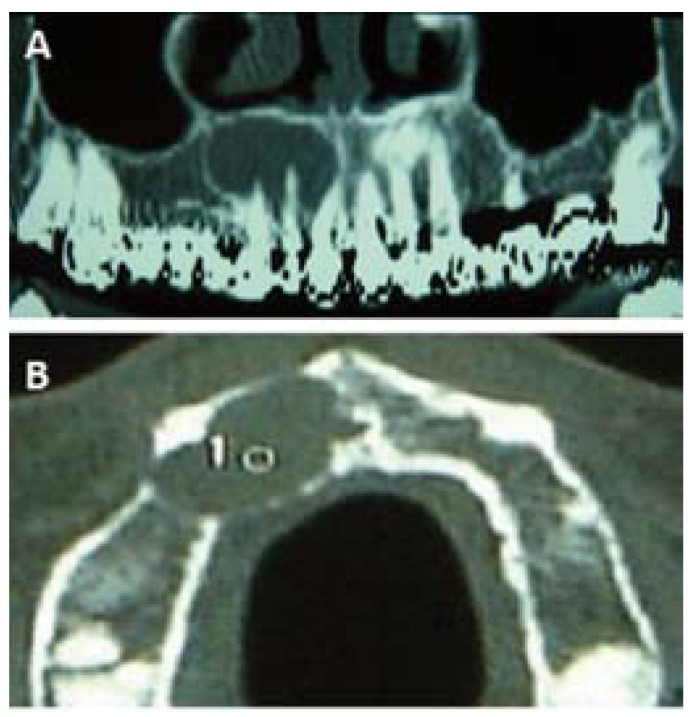
A) Tomographic reconstruction of the upper maxilla: Radiotransparency on the right side, displacing the floor of the nasal passages. B) CT. Axial view: Bulging of the vestibular and palatine cortical layers.

A 55-year-old Caucasian woman was attended in the Service of Maxillofacial Surgery for evaluation and treatment of a right upper maxillary lesion affecting canine, lateral and central incisors (teeth #6, #7 and #8). Local clinical examination and on the rest of the orofacial structures was unremarkable. The radiographic study (Fig. 1) showed a circular radiotransparency with sclerotic margins enveloping the apexes of canine, lateral and central incisors (teeth #6, #7 and #8) – all of which had undergone endodontic treatment several years ago. In the tomographic reconstruction images of the maxilla, the lesion had a low bone density appearance, close to the floor of the nasal passages and a slight bulging of the buccal and palatal cortical layer – the latter presenting discontinuous margins (Fig. 2).

- Differential diagnosis

The present lesion was identified on occasion of a radiographic study of the abutment teeth of maxillary dentures. The patient history and clinical examination yielded no data suggestive of the presence of an odontogenic abscess. No surgery had been performed in the area that would suggest a possible residual cyst or apical scar. The presumed diagnosis was therefore a radicular cyst associated to any of these teeth. On the basis of the location of the lesion, the X-ray study and diagnostic study images, the differential diagnosis included lateral periodontal cyst, keratocystic odontogenic cyst, ossifying fibroma, and other less common odontogenic tumors.

- Diagnosis and treatment

**Figure 3:**
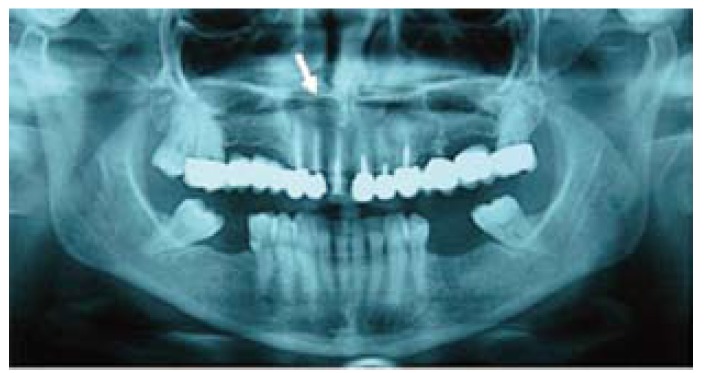
Panoramic radiograph. Postoperative control at 12 months.

The lesion was removed, with periapical surgery of the affected teeth ( #6, #7 and #8) under local anesthesia and conscious sedation. Surgical specimen measured 1 x 1 cm. The patient is currently under periodic clinical and radiological controls in order to detect any possible relapse (Fig. 3).

**Figure 4:**
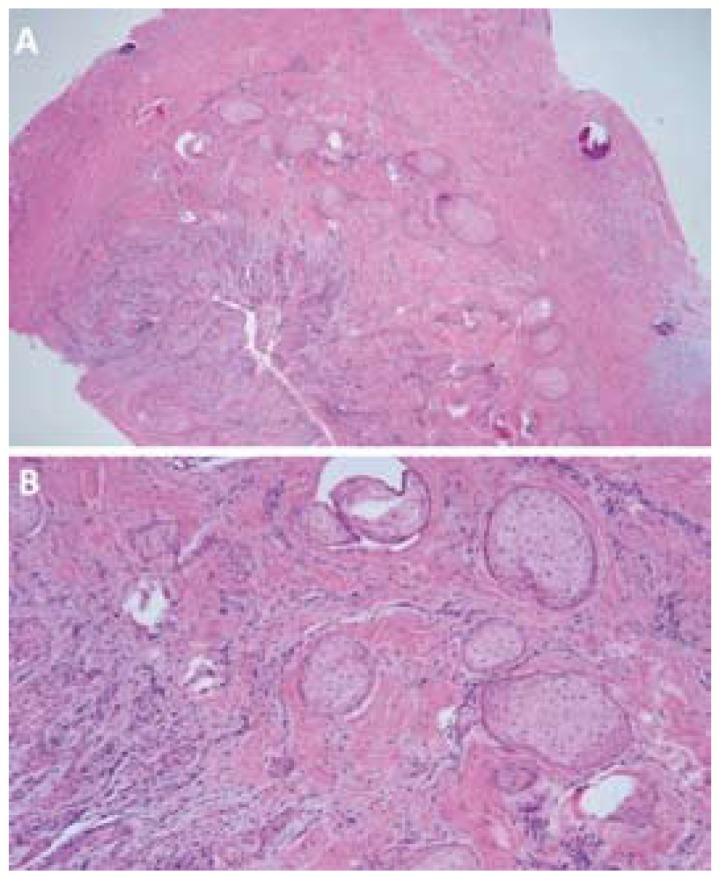
Histological section of the SOT-like proliferation. A) Epithelial cell aggregates. Hematoxylin-eosin stain, x40. B) Hematoxylin-eosin stain, x100.

- Histopathologic analysis:

After staining with hematoxylin-eosin, the specimen was examined under the light microscope at x40 and x100 magnification (Fig. 4). The histological study revealed a cyst lined by hyperplastic squamous epithelium with a chronic inflammatory infiltrate of the fibrous wall and a series of squamous odontogenic cell proliferations forming rounded aggregates surrounded by the fibrous tissue. Based on these findings, a diagnosis of radicular cyst with proliferating squamous odontogenic epithelium was established. ( Table 1)

**Table 1:**
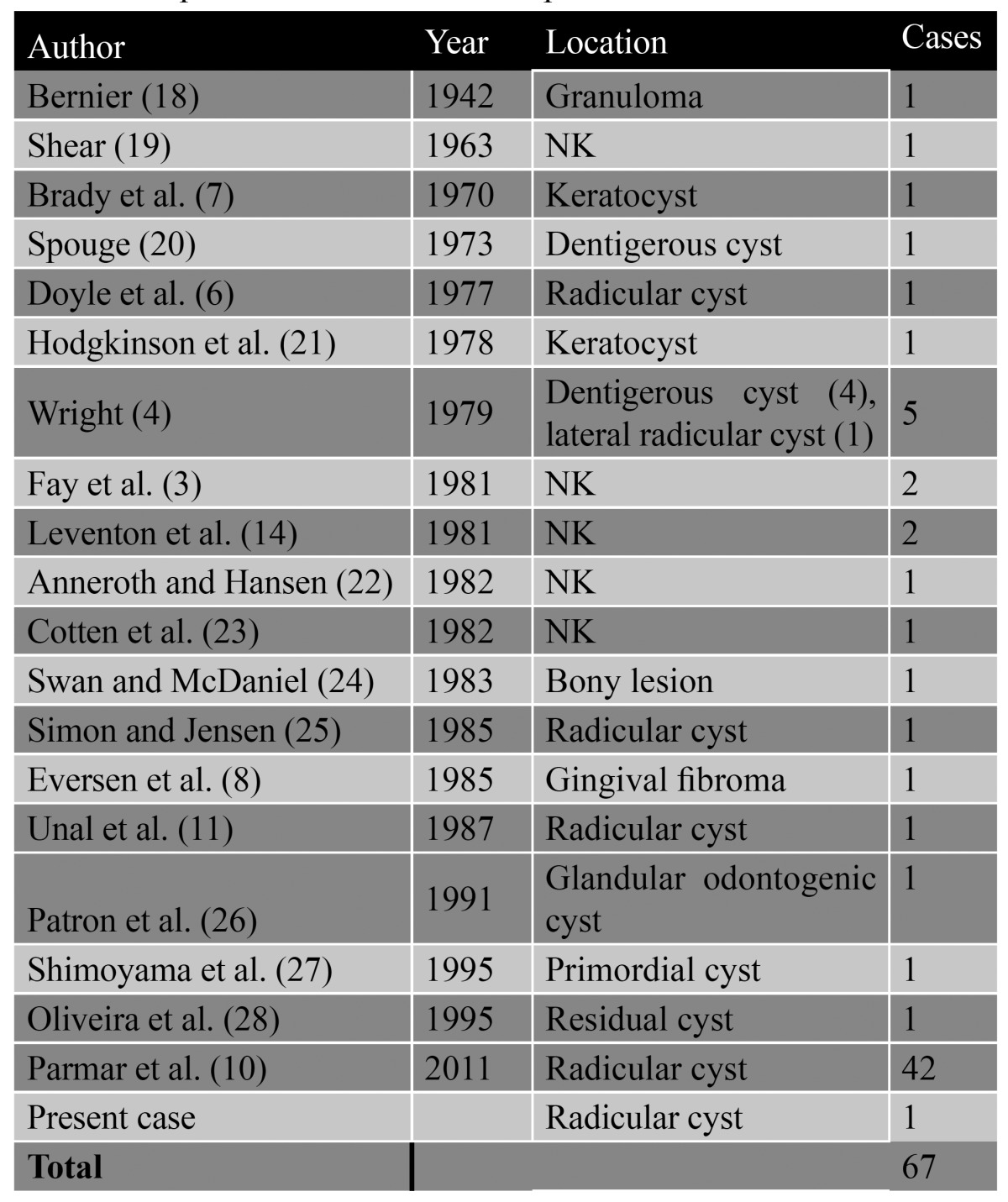
Reported cases of SOT- like proliferation. NK = not known

## Discussion

Squamous odontogenic tumor (SOT) is an infrequent, benign maxillary neoplasm originating from the epithelial rests of Malassez of the periodontal ligament. It is a slow and progressively growing lesion that invades the trabecular bone and can deform and perforate the cortical layers (central variant) or erode the alveolar crest (peripheral variant), inducing mobility of the remaining teeth with swelling and moderate pain of the gums (4). However, a non-neoplastic lesion with the same characteristics also exists, in the form of a squamous odontogenic tumor-like proliferation (SOTLP), which is discussed in relation to our case. This entity was first described in 1975 by Pullon et al. (1), who published a series of 6 cases. However, two years later Doyle et al. (6) described the first case of a SOTLP within the thickness of the membrane of a radicular cyst. Until that time, all published cases had been regarded as latent or incipient neoplasms. Goldblatt et al. (2) disagreed with this view and suggested that the absence of cellular atypia in SOTLP indicates that such lesions are not true neoplasms. The etiology is not known although some authors consider them to develop from the rests of Serres, or even from the epithelial rests of Malassez within the periodontal ligment (1,6). Parmar et al. (3) observed in their review of 42 cases of SOTLP that the presence of hyperplasia in the epithelial cyst lining could also contribute to its growth. These epithelial rests derive from the embryonic remains generated in the course of odontogenesis, and remain latent and isolated for long periods in the different maxillary structures. They in turn are able to reactivate, grow and give rise to epithelial odontogenic tumors, or remain dormant. Bouquot et al. (7) documented a total of 84 epithelial rests and 5 incipient odontogenic tumors (one ameloblastoma, two SOTs and two calcifying epithelial odontogenic tumors) related to the periodontal ligament in both the upper maxilla and the mandible. Another possible origin of these tumors is the pericoronal follicle of unerupted teeth, or of impacted teeth (8). The most frequent location of SOTLP is the membrane of an odontogenic cyst (radicular cyst (6), keratocystic odontogenic tumor (9), follicular cyst or residual cyst (10)), as in our case – though they have also been identified at intramaxillary level, in an isolated cavity and in a pediculate mass of fibrous tissue at gingival level (11). SOT and SOTLP are histologically very similar, with the presence in both cases of aggregates of well differentiated and non-keratinized squamous odontogenic epithelial cells surrounded by fibrous connective tissue. In our case, the histological study of the lesion revealed an isolated structural alteration within the membrane of a radicular cyst, with proliferating squamous odontogenic epithelial cells, and no signs of cellular atypia. The squamous cell aggregates that conform this condition in an odontogenic cyst are morphologically different from the rest of developing epithelial cells that form part of the cyst membrane. Perhaps for this reason they have been suggested to conform genuine hamartomatous structures (2). The growth of such lesions is usually limited, and they generate no symptoms. Radiologically, SOTs are solitary, unilocular and radiotransparent images, though multilocular presentations with involvement of the entire mandibular body are also possible. In some cases displacement of the membrane of the maxillary sinus can be observed. The lesions appear alongside erupted permanent teeth, in edentulous regions, and exceptionally affect the primary dentition (4). In our case this entity manifested as a unilocular radiotransparency around the apexes of three devitalized teeth, with no associated tooth displacement or root reabsorption. The vestibular and palatine cortical layers were slightly bulged, and there was no invasion of the maxillary sinus or displacement of the nasal passages. Since SOTLP may have possibly the same origin as SOTs (the epithelial rests of Malassez), it has been suggested that this entity could be the initial expression of the neoplastic form (12) – though to date no neoplastic changes or signs of malignancy have been documented. The malignant transformation of an odontogenic cyst is a rare event, with an incidence of 1-2%. Most such cases correspond to primary intrabony squamous cell carcinomas. Müller and Waldron (13) in a review of 119 such carcinomas, found 70% to originate within an odontogenic cyst. On the other hand, in a review of 4172 maxillary cysts, Timosca et al. (14) only documented 5 cases (0.12%) of malignant transformation. The odontogenic cysts with the greatest malignization potential include radicular/residual cysts, lateral periodontal cysts and keratocystic odontogenic tumors – though a case of SOT transforming into squamous cell carcinoma has also been reported (15). Based on the above mentioned, we consider essential to carry out a thorough histological study of all lesions removed from the oral tissues. In the presence of a SOTLP, it is very important not to establish a wrong diagnosis of SOT or other conditions such as ameloblastoma or primary intrabony squamous cell carcinoma. The management of this condition comprises enucleation, curettage or local excision of the lesion (4). To date, there have been no reports of recurrences or malignant changes in the literature relating to odontogenic cysts with SOTLP. Nevertheless, frequent clinical and radiological controls are required with the purpose of identifying possible neoplastic changes and lesion recurrences.
